# Supplementary material for: Substitutions into amino acids that are pathogenic in human mitochondrial proteins are more frequent in lineages closely related to human than in distant lineages
Source: PeerJ. 2017 Dec 12;5:e4143. doi: 10.7717/peerj.4143 (PMC5731343; doi:10.7717/peerj.4143)
Supplement: Supplemental Information 2 [file peerj-05-4143-s002.docx]

**Table S1.** Overlapping between two datasets

| **Gene** | **Species in**  **metazoan dataset** | **Species in**  **opisthokonts dataset** | **Common species** |
| --- | --- | --- | --- |
| ATP6 | 2931 | 4350 | 1295 |
| COX1 | 4366 | 4350 | 900 |
| COX2 | 4131 | 4350 | 854 |
| COX3 | 2152 | 4350 | 1331 |
| CYTB | 5995 | 4350 | 1436 |

**Table S2.** Homoplasies giving rise to the human reference allele.

| **Gene** | **Species** | **Sites** | **Analyzed sites** | **Substitutions to the human amino acid per site** | **Substitutions to the human amino acid per site in simulation** |
| --- | --- | --- | --- | --- | --- |
| ATP6 | Metazoans | 186 | 131 | 25.3 | 16.7 |
| COX1 | Metazoans | 404 | 146 | 12.1 | 13.9 |
| COX2 | Metazoans | 165 | 110 | 22.5 | 22.3 |
| COX3 | Metazoans | 198 | 128 | 22.4 | 16.2 |
| CYTB | Metazoans | 327 | 183 | 32.2 | 29.7 |
| ND1 | Metazoans | 253 | 160 | 15.5 | 12.1 |
| ND2 | Metazoans | 299 | 220 | 36.3 | 34.0 |
| ND3 | Metazoans | 94 | 67 | 26.6 | 20.0 |
| ND4 | Metazoans | 392 | 263 | 19.9 | 17.3 |
| ND4L | Metazoans | 82 | 70 | 18.4 | 19.6 |
| ND5 | Metazoans | 516 | 302 | 8.7 | 7.3 |
| ND6 | Metazoans | 119 | 91 | 11.4 | 10.1 |
| ATP6+COX1+COX2+COX3+CYTB | Opisthokonts | 1524 | 964 | 30.2 | 24.4 |

**Table S3.** Homoplasies giving rise to the human non-reference allele.

| **Gene** | **Species** | **Polymorphic sites** | **Analyzed sites** | **Substitutions to the human non-reference allele per non-reference allele** | **Mean no. of non-reference alleles per polymorphic site** |
| --- | --- | --- | --- | --- | --- |
| ATP6 | Metazoans | 142 | 128 | 21.1 | 1.9 |
| COX1 | Metazoans | 131 | 104 | 21.0 | 1.4 |
| COX2 | Metazoans | 81 | 71 | 28.3 | 1.4 |
| COX3 | Metazoans | 108 | 92 | 24.8 | 1.6 |
| CYTB | Metazoans | 199 | 187 | 36.0 | 1.7 |
| ND1 | Metazoans | 101 | 87 | 16.8 | 1.5 |
| ND2 | Metazoans | 143 | 129 | 49.1 | 1.6 |
| ND3 | Metazoans | 34 | 32 | 30.3 | 1.6 |
| ND4 | Metazoans | 136 | 109 | 20.9 | 1.2 |
| ND4L | Metazoans | 29 | 25 | 17.3 | 1.5 |
| ND5 | Metazoans | 223 | 171 | 9.9 | 1.6 |
| ND6 | Metazoans | 55 | 45 | 11.1 | 1.5 |
| ATP6+COX1+COX2+COX3+CYTB | Opisthokonts | 775 | 516 | 32.2 | 1.6 |

**Table S4.** Homoplasies giving rise to the human pathogenic variants.

| **Gene** | **Species** | **Sites with pathogenic alleles** | **Analyzed sites** | **Substitutions to**  **the human pathogenic amino acid per pathogenic amino acid** | **Mean no. of pathogenic amino acids per site with pathogenic alleles** |
| --- | --- | --- | --- | --- | --- |
| ATP6 | Metazoans | 15 | 11 | 9.7 | 1.1 |
| COX1 | Metazoans | 20 | 15 | 41.0 | 1.0 |
| COX2 | Metazoans | 10 | 9 | 44.8 | 1.0 |
| COX3 | Metazoans | 9 | 7 | 14.8 | 1.0 |
| CYTB | Metazoans | 20 | 16 | 15.5 | 1.1 |
| ND1 | Metazoans | 25 | 17 | 6.3 | 1.1 |
| ND2 | Metazoans | 10 | 8 | 29.5 | 1.0 |
| ND3 | Metazoans | 6 | 5 | 3.5 | 1.0 |
| ND4 | Metazoans | 7 | 4 | 15.1 | 1.2 |
| ND4L | Metazoans | 3 | 3 | 6.0 | 1.0 |
| ND5 | Metazoans | 24 | 8 | 5.1 | 1.0 |
| ND6 | Metazoans | 13 | 10 | 8.0 | 1.1 |
| ATP6+COX1+COX2+ COX3+CYTB | Opisthokonts | 89 | 72 | 25.0 | 1.0 |
